# Inhibition of DEK Enhances Doxorubicin-Induced Apoptosis and Cell Cycle Arrest in T-Cell Acute Lymphoblastic Leukemia Cells

**DOI:** 10.1155/2022/9312971

**Published:** 2022-06-20

**Authors:** Xiaoxue Tian, Zeyu Zhu, Guangming Wang, Jun Xu, Aibin Liang, Wenjun Zhang

**Affiliations:** ^1^Department of Hematology, Tongji Hospital, Tongji University School of Medicine, 1239 Siping Road, Shanghai 200092, China; ^2^East Hospital, Tongji University School of Medicine, 1239 Siping Road, Shanghai 200092, China

## Abstract

T-cell acute lymphoblastic leukemia (T-ALL) is a serious hematological tumor derived from early T-cell progenitors, which is extremely resistant to chemotherapy. Classically, doxorubicin (DOX) is an effective first-line drug for the treatment of T-ALL; however, DOX resistance limits its clinical effect. The DEK proto-oncogene (DEK) has been involved in neoplasms but remains unexplored in T-ALL. We silenced DEK on Jurkat cells and detected cell proliferation with cell counting and colony formation assay. Then, we detected DEK's drug sensitivity to DOX with CCK-8, cell cycle, and apoptosis with DOX treatment. Western blot analysis was performed to determine protein expression of apoptosis and cell cycle-related genes, including BCL2L1, caspase-3, and cyclin-dependent kinases (CDK). Finally, the tumorigenic ability of DEK was analyzed using a BALB/C nude mouse model. In this study, DEK was highly expressed in Jurkat cells. Inhibition of DEK can lead to decreased cell proliferation and proportion of S-phase cells in the cell cycle and more cell apoptosis, and the effect is more obvious after DOX treatment. Western blot results showed that DOX treatment leads to cell cycle arrest, reduction of cyclin-dependent kinase 6 (CDK6) protein, accumulation of CDKN1A protein, and DOX-induced apoptosis accompanied by reductions in protein levels of BCL2L1, as well as increases in protein level of caspase-3. Furthermore, DEK-silenced Jurkat cells generated a significantly smaller tumor mass in mice. Our study found that DEK is a novel, potential therapeutic target for overcoming DOX resistance in T-ALL.

## 1. Introduction

T-cell acute lymphoblastic leukemia (T-ALL) is a serious hematological tumor that is metastatic, aggressive, and resistant to chemotherapy [1], accounting for approximately 15% of ALL cases in children and 25% in adults [2]. With the advances in induction therapy, the event-free survivals of T-ALL patients have exceeded 85% in recent clinical trials [3]. However, about 20% of children and 40% of adults with T-ALL will relapse after intensive chemotherapy, leading to a 5-year overall survival of 50%–60% [4]. Chemoresistance is considered a major cause of recurrence and death of T-ALL [5]. Thus, resensitizing drug-resistant leukemia cells to chemotherapy may improve the prognosis of T-ALL patients.

Recently, the systematic gene expression has been emphasized [6]. The DEK proto-oncogene (DEK) is preferentially expressed in malignant cells [7]. DEK facilitates the tumorigenesis of different types of cancer cells by promoting cell proliferation and modulating cell cycle transition, as well as inhibiting cell apoptosis and senescence [8]. Furthermore, apoptosis induced by DEK deletion was accompanied by an increase in TP53 activity and its upregulation of CDKN1A and Bax [9]; this effect may be related to growth retardation and activation of TP53 function. CDKN1A mediates cell cycle arrest in the G1 and G2 phase and leads to cell apoptosis, and it can effectively inhibit CDK2, CDK3, CDK4, and CDK6 [10–12]. In melanoma, the downregulation of DEK significantly increased cell apoptosis and senescence through DOX treatment and had no effect on TP53 and CDKN2A levels but had a significant effect on CDKN1A and caspase-3 levels [13]. DEK overexpression has been seen in many neoplasms, including chronic lymphocytic leukemia and acute myeloid leukemia [14, 15]. However, the involvement of DEK in T-ALL remains unexplored. It has been reported that DEK silencing may increase cancer cell sensitivity to DOX treatment in nonsmall cell lung cancer and metastatic colorectal cancer [16, 17]. Thus, we hypothesized that DEK silencing might enhance the sensitivity of leukemia cells.

Doxorubicin (DOX) is an anthracycline chemotherapeutic agent that is commonly used to treat ALL [18, 19]. Anthracyclines such as DOX, a topoisomerase II, kill leukemia cells by inhibiting cellular RNA and DNA synthesis [20, 21]. However, the efficacy of DOX is limited by the development of chemoresistance in leukemia cells [22]. DEK deficiency in different tumor cells has been shown to increase their sensitivity to DOX [13, 20]. Based on these studies, we supposed that the downregulation of DEK can enhance the sensitivity of Jurkat cells to DOX chemotherapy in T-ALL cells.

In this study, we determined DEK expression in different leukemia cell lines and found that DEK is highly expressed in Jurkat cells. Thus, we inhibited DEK expression in Jurkat cells to investigate the role and the underlying mechanism of DEK in the cellular response to DOX. We also explored the role of DEK in the tumorigenicity of Jurkat cells in a murine model. Our results suggest that DEK silencing may increase the sensitivity of Jurkat cells to DOX treatment, serving as a promising therapeutic approach for the management of DOX-resistant T-ALL.

## 2. Materials and Methods

### 2.1. Cell Lines

293T, Raji, SU-DHL-4, Daudi, Nalm6, Jurkat, Panc-1,U937, PC-3, and MCF-7 cell lines (Shanghai Cell Bank). High glucose DMEM (SH30022.01B, Hyclone) was used to culture the 293T, Panc-1, and MCF-7 cell line. The remaining hematological tumor cell lines were cultured in RPMI-1640 medium (SH30809.01B, Hyclone). All cell lines were incubated at 37°C with 5% CO_2_.

### 2.2. Gene Knockdown

shRNAs targeting DEK and negative control (scramble, SCR) vectors were purchased from Genomeditech. The shRNA sequences were as follows: shDEK-1, 5′-GCCAGTGCTAACTTGGAAGAA-3′; shDEK-2, 5′-GCCTGAAATTCTGTCAGATGAA-3′; and Scramble, 5′-GTTCTCCGAACGTGTCACGT-3′. The Jurkat cell line was infected with lentiviral supernatant and then analyzed *in vitro* for proliferation, cell viability, colony formation, cell cycle, and apoptosis.

### 2.3. RT-PCR

Total RNA was extracted from Jurkat cells at 48 h after transduction, using a Quick-RNA™ Microprep Kit (Zymo, Irvine, CA, USA). PCR was performed on a LightCycler 96 PCR system (Roche Life Science, Indianapolis, IN, USA). The primers were as follows: GAPDH, forward, 5′-CTCTGATTTGGTCGTATTGGG-3′, and reverse, 5′-TGGAAGATGGTGATGGGATT-3′; DEK, forward, 5′-AACTGCTTTACAACAGGCCAG-3′, and reverse, 5′-ATGGTTTGCCAGAAGGCTTTG-3′. The relative expression of DEK was calculated using the 2^-*ΔΔ*Ct^ method [24].

### 2.4. Colony Formation Assay

Jurkat cells were seeded into a 12-well plate coated with agarose (1.2% at the bottom and 0.6% on the top) at a density of 1 × 10^3^ cells per well and transduced with lentiviral vectors expressing scramble shRNA or shDEK. After 14 days of culture, the number of colonies was counted at a magnification of 4x using an inverted microscope (AE2000; Motic, China).

### 2.5. Cell Counting Kit-8 (CCK-8) Assay

Jurkat cells were seeded in a 96-well plate at 5 × 10^3^ cells per well and transduced with lentiviral vectors expressing scramble shRNA or shDEK. Cell viability was determined at 72 h after transduction using CCK-8 (Dojindo, Japan). Then, a microplate reader was used at an optical density of 450 nm.

### 2.6. Cell Apoptosis Analysis

We seed 1 × 10^6^ cells per well in a 6-well plate and grow them at 37°C in a medium containing DOX or PBS for 4 hours. Then, the cells were washed 3 times with PBS and continued to be cultured in a cell incubator. Cells were washed 3 times with PBS and collected, then resuspended in 100 *μ*l 1x binding buffer, stained with annexin V-APC at room temperature for ten minutes, and then stained with propidium iodide (PI) at room temperature for 5 minutes in the dark (BD Biosciences).

### 2.7. Cell Cycle Analysis

Bromodeoxyuridine (BrdU, BD biosciences, USA) and PI double staining was performed to detect cell cycle distribution. 1 × 10^6^ cells were seeded and incubated with 3 *μ*g/ml BrdU for 2 hours in 6-well plates. Cells were then harvested, mixed with 70% ethanol, and fixed overnight at -20°C. Samples were treated according to APC-BrdU antibody (BioLegend), and PI solution was added 5 minutes before flow cytometry analysis.

### 2.8. Western Blotting

Jurkat cells were harvested 5 days after lentiviral infection after transduction and lysed in RIPA lysis buffer (PC101, Epizyme Biotech). Then, the protein samples were mixed with 1x SDS (LT101S; Epizyme Biotech), boiled for 10 minutes, and then subjected to PAGE gel electrophoresis. The primary antibody used in the experiment includes DEK (E4S5J; Cell Signaling Technology), GAPDH (D16H11; Cell Signaling Technology), TP53 (DO-7; Cell Signaling Technology), c-Myc (ab32072; Abcam), CDK4 (A11136; ABclonal), CDK6 (13331; Cell Signaling Technology), CDKN1A (A1483; ABclonal), CDKN2A (ab151303; Abcam), caspase-3 (9662; Cell Signaling Technology), BCL2L1 (A19703; ABclonal) at 4°C, and HRP-conjugated secondary antibody (anti-rabbit,7074S, anti-mouse; 7076S,Cell Signaling Technology) at room temperature for 2 h. The target protein was detected by using Omni-ECL™-enhanced chemiluminescent liquid (SQ101; Epizyme Biotech) and quantified using ImageQuant LAS 4000 mini (GE).

### 2.9. Animal Model

10^7^ Jurkat cells from the SCR group or DEK knockdown (KD) group were injected into the subcutaneous tissue of female adult BALB/c nude mice in a volume of 100 *μ*l for *in vivo* tumor growth studies. Thirty days after transplantation, euthanizing mice in each group, the tumor volume was calculated as follows: tumor volume = length × (width^2^)/2, and tumor sizes were analyzed [23]. All animal experiments were performed in accordance with the standards of Tongji University School of Medicine.

### 2.10. Statistical Analysis

All quantitative data are displayed as mean ± SEM, and analyses were executed using Prism 8.0.

Unpaired two-tailed Student's *t*-test is used for data analysis. FCS Express 10 Flow software analyzes flow cytometry data. Differences were considered statistically significant at *P* < 0.05.

## 3. Results

### 3.1. DEK Is Highly Expressed in Jurkat Cells

**Figure 1:**
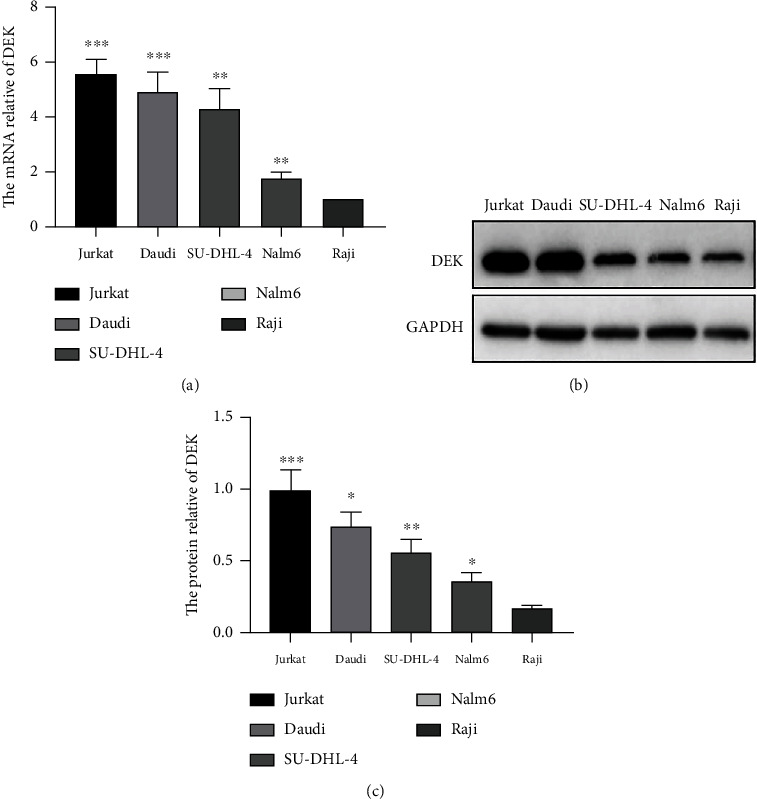
DEK is highly expressed in Jurkat T-ALL cells. (a) DEK mRNA expression in Jurkat, Daudi, Nalm6, SU-DHL-4, and Raji cells was analyzed by RT-PCR. (b) Protein expression levels of DEK and GAPDH in Jurkat, Daudi, Nalm6, SU-DHL-4, and Raji cells. (c) Quantification of DEK protein level by densitometric analysis. ^∗^*P* < 0.05, ^∗∗^*P* < 0.01, and ^∗∗∗^*P* < 0.001.

To determine DEK expression in leukemia, assays were performed in different leukemia cell lines using RT-PCR and western blotting. The Raji cell line expressing the lowest DEK was selected as a control among the acute leukemia and lymphoma cell lines tested. The Jurkat cell line showed the highest level of DEK mRNA and protein (Figure 1). Of these cell lines, these results suggested that DEK is highly involved in T-ALL development. Results of the human protein analysis (https://www.proteinatlas.org/ENSG00000124795-DEK/tissue) showed the level of DEK mRNA transcripts in different cancer cell lines and normal tissues (Supplemental Figure S1). Therefore, experiments for DEK phenotypic and functional validation were performed using Jurkat cells.

### 3.2. shRNA-Mediated DEK Knockdown Efficiently Suppresses Cell Proliferation

**Figure 2:**
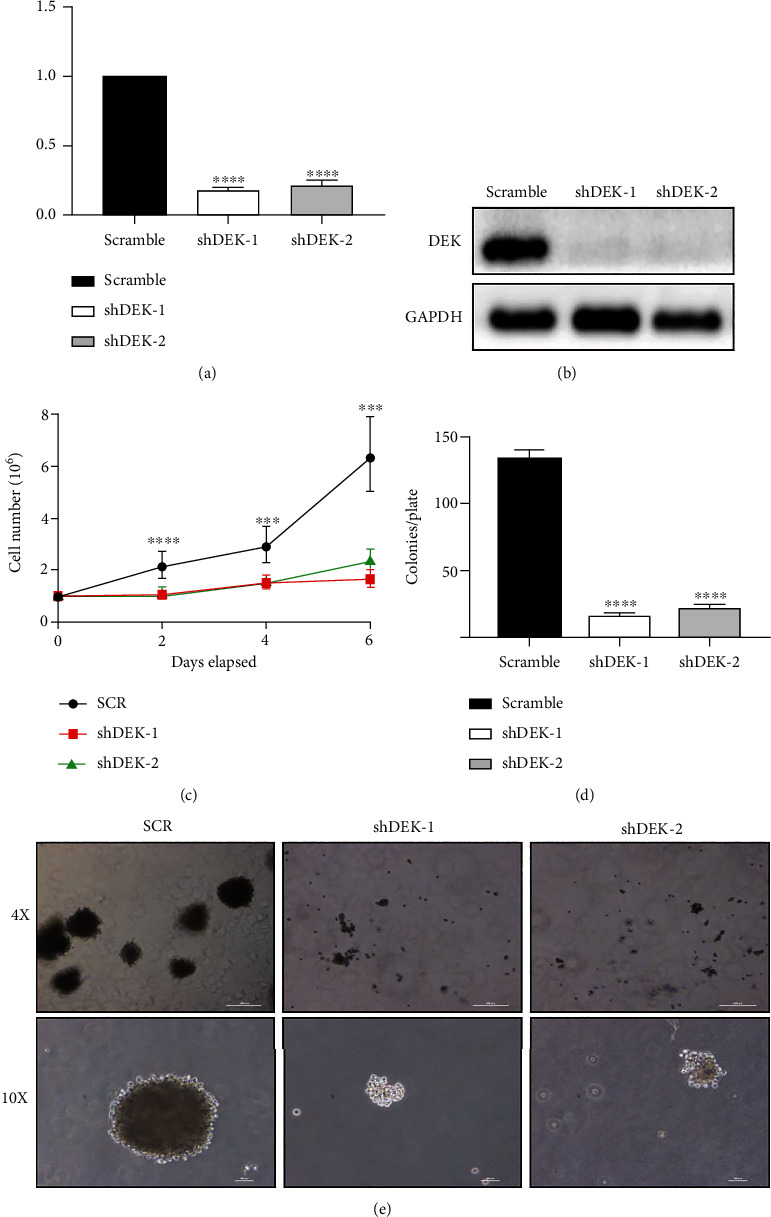
DEK silencing efficiently suppresses cell proliferation. (a) DEK mRNA levels relative to GAPDH levels in Jurkat cells infected with three different lentiviruses (SCR, shDEK-1, and shDEK-2) as detected by RT-PCR. (b) Western blotting was conducted to confirm that shDEK efficiently knocked down DEK protein expression in Jurkat cells. (c) Cell proliferation assay. Cell numbers were counted at 0, 2, 4, and 6 days after transduction. Data are expressed as the mean ± SEM. ^∗∗∗^*P* < 0.001 vs. SCR; *n* = 3. (d) Colony formation assay. The number of colonies formed by Jurkat cells was counted at 14 days after transduction. (e) Representative images of colonies formed by DEK KD Jurkat cells after 14 days. ^∗∗∗^*P* < 0.001 and ^∗∗∗∗^*P* < 0.0001.

We used the DEK-KD group and SCR group to conduct cell proliferation experiments in Jurkat cell. As shown in Figures 2(a) and 2(b), shDEK effectively suppressed DEK mRNA and protein expression of Jurkat cells compared with scramble shRNA. The cell proliferation assay showed that knockdown of DEK significantly inhibited Jurkat cell proliferation compared with SCR group starting 2 days after transduction (day 2: *P* < 0.0001, day 4 and day 6: *P* < 0.001; Figure 2(c)). Colony formation assay showed that the number of colonies formed by DEK-silenced cells was dramatically less than the number of colonies formed by the SCR group (28 ± 6 and 39 ± 4 vs. 135 ± 7; *P* < 0.0001; Figure 2(d)). Consistent results were observed in the size of colonies (Figure 2(e)). These data suggest that knockdown of DEK suppresses leukemia cell proliferation and colony formation. Thus, DEK is a novel target of T-ALL treatment.

### 3.3. DEK Inhibition in Jurkat Cells Increases the Response to DOX

**Figure 3:**
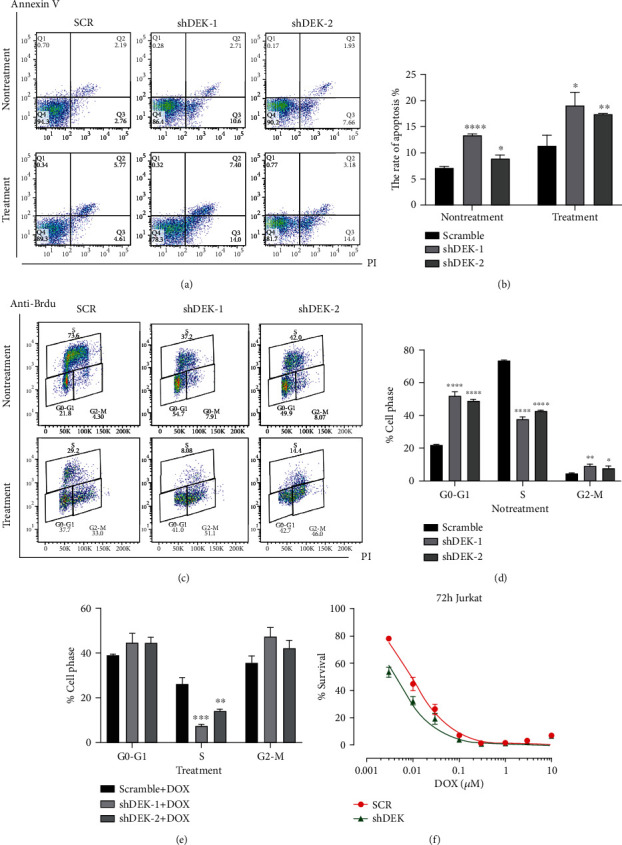
Knockdown of DEK promotes doxorubicin- (DOX-) induced apoptosis and cell cycle arrest of Jurkat cells. (a, b) Cells were treated with vehicle or DOX for 72 h at 5 days after lentiviral infection, and then, we examined cell apoptosis via flow cytometry. (c–e) Flow cytometry analysis was carried out to examine cell cycle phase distribution of Jurkat cells. (f) Cell viability in SCR and DEK KD groups was detected by CCK-8. Data are expressed as the mean ± SEM. ^∗^*P* < 0.05, ^∗∗^*P* < 0.01, ^∗∗∗^*P* < 0.001, and ^∗∗∗∗^*P* < 0.0001; shDEK-1 and shDEK-2 vs. SCR or shDEK-1+DOX and shDEK-2+DOX vs. SCR+DOX; *n* = 3. SCR: scramble RNA.

We treated SCR Jurkat cells and DEK-silenced Jurkat cells with DOX and then performed cell viability, apoptosis, and cell cycle distribution. The results of CCK-8 analysis further showed that compared with the negative control, knockdown of DEK significantly reduced the cell viability of Jurkat cells in the presence of DOX ranging from 0 to 10 *μ*M (IC50 of SCR group: 9.306 nM, IC50 of shDEK group: 3.744 nM; Figure 3(f)). The apoptotic rates of Jurkat cells in the DEK-KD groups were 13.02 ± 0.58% and 9.53 ± 0.91%, compared with 4.95 ± 0.41% in the SCR group as shown in Figures 3(a) and 3(b) (shDEK-1: *P* < 0.05, shDEK-2: *P* < 0.0001). Following DOX treatment, the apoptotic rates of DEK KD groups were 19.3 ± 0.49% and 17.58 ± 0.23% compared with 10.38 ± 0.92% in the SCR group (shDEK-1: *P* < 0.05, shDEK-2: *P* < 0.01; Figures 3(a) and 3(b)). In brief, these results proved that DEK silencing increased the induction of apoptosis via DOX in Jurkat cells.

BrdU is a synthetic thymidine analog that is incorporated during the S phase of cellular DNA replication [25]. After the DNA is denatured, the cells are stained to allow BrdU incorporation, and any other target cell surfaces and/or intracellular targets are stained. The rates of S-phase cells in the DEK KD Jurkat cells were 37.67 ± 1.53% and 42.53 ± 0.47% versus 73.3 ± 0.73% in the SCR cells, the rates of G0/G1 phase cells in the DEK KD groups were 52.03 ± 2.67% and 48.93 ± 0.83% versus 21.83 ± 0.36% in the SCR cells in Figures 3(c) and 3(d) (^∗∗∗∗^*P* < 0.0001), and the proportions of G2/M-phase cells in the DEK KD groups were 9.06 ± 1.53% and 7.67 ± 1.61% versus 4.47 ± 0.17% in the SCR Jurkat cells (shDEK-1: *P* < 0.01, shDEK-2: *P* < 0.05). With DOX treatment, the proportions of S-phase cells were 7.41 ± 0.47% and 14.1 ± 0.9% in the KD groups and 26 ± 2.9% in the SCR group (Figures 3(c) and 3(e), shDEK-1: *P* < 0.001, shDEK-2: *P* < 0.01). These results indicate that under normal growth conditions, DEK silencing leads to reduced cell distribution in the S phase, cell arrest in the G0/G1 phase, and cell cycle arrest in the G2/M phase with DOX treatment.

### 3.4. DEK Regulates Apoptosis and Cell Cycle-Related Genes

**Figure 4:**
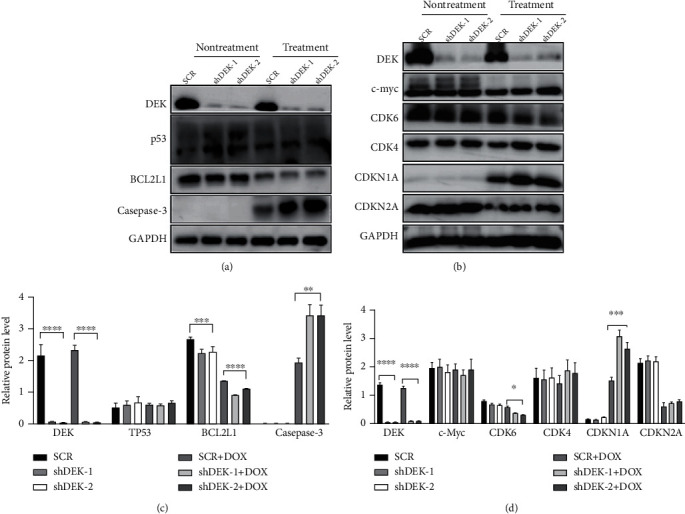
Expression of apoptosis- and cell cycle-related proteins in Jurkat cells. (a, b) Western blot analysis was conducted to measure the protein levels of SCR, shDEK-1, or shDEK-2 as indicated. (a, b) Representative blots are shown. (c) Quantification of (a). (d) Quantification of (b). GAPDH was used as an internal control. Data are expressed as the mean ± SEM. ^∗^*P* < 0.05, ^∗∗^*P* < 0.01, ^∗∗∗^*P* < 0.001, ^∗∗∗∗^*P* < 0.0001 vs. SCR; *n* = 3. SCR: scramble RNA.

The contribution of DEK in cancer progression involves the alterations in TP53, CDKN1A, c-Myc, and other apoptosis- and cell cycle-related genes [13, 26]. In melanoma, DEK silencing considerably increased cell apoptosis and senescence through DOX treatment and had no effect on TP53 and CDKN2A levels but had a significant effect on CDKN1A and caspase-3 levels [13]. As shown in Figures 4(a)–4(d), DEK silencing did not affect the protein expression of TP53, c-Myc, or CDKN2A regardless of the presence or absence of DOX, compared with SCR group. However, DEK silencing significantly suppressed BCL2L1 protein expression under normal conditions (*P* < 0.001) and further attenuated BCL2L1 protein expression repressed by DOX (*P* < 0.0001). In contrast, knockdown of DEK further enhanced DOX-induced caspase-3 protein expression (*P* < 0.01; Figures 4(a) and 4(c)). Regarding cell cycle-related genes, knockdown of DEK significantly suppressed CDK6 expression in the presence of DOX, respectively, compared with the SCR group (both *P* < 0.05). DEK silencing also further enhanced DOX-induced upregulation of CDKN1A expression (*P* < 0.001; Figures 4(b) and 4(d)). These data suggest that DEK silencing enhances the DOX sensitivity of Jurkat cells by modulating some apoptosis- and cell cycle-related genes in a TP53/CDKN2A/c-Myc-independent manner.

### 3.5. DEK Silencing Reduces the Tumorigenesis Ability of Jurkat Cells

**Figure 5:**
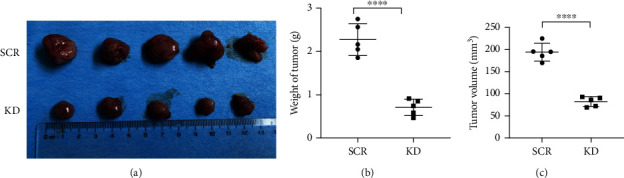
*In vivo* tumorigenesis ability study. Knockdown of DEK suppressed the growth of Jurkat cell-derived tumors in mice. (a) Image of tumors derived from Jurkat cells. (b, c) Tumor weights and volumes at 30 days after inoculating Jurkat cells transduced with negative control or shDEK. ^∗∗∗∗^*P* < 0.0001 vs. SCR, *n* = 5. SCR: scramble RNA; KD: knockdown.

To investigate the effect of DEK silencing in vivo, we established a tumor model by subcutaneously injecting DEK-silenced Jurkat cells or control cells into female adult BALB/C nude mice. The tumor volume in the DEK KD group was 82 ± 13 mm^3^ and the tumor weight was 0.708 ± 0.248 g, whereas the tumor volume in the SCR group was 194.4 ± 24.4 mm^3^ and the tumor weight was 2.28 ± 0.42 g (Figures 5(a)–5(c), ∗∗∗∗*P* < 0.0001). The DEK KD mice were less aggressive and showed smaller tumor sizes than the mice we injected with SCR Jurkat cells.

## 4. Discussion

T-ALL is a serious hematological tumor and is highly resistant to chemotherapy, occurs in both adults and children, and has a high rate of recurrence [27, 28]. DEK plays a potential role in hematopoiesis and is dysregulated in acute myeloid leukemia and chronic lymphocytic leukemia [14, 15]; however, the involvement of DEK in T-ALL remains unknown.

Many studies have focused on the expression of cytokines [29]. Of note, it has been reported that DEK is overexpressed in most tumors of different origins, and tumorigenesis is promoted by promoting cell self-renewal and proliferation while inhibiting apoptosis, differentiation, and senescence of malignant cells [8, 9]. DEK-targeted inhibition has been considered as an effective treatment strategy of different malignancies due to its frequent upregulation in human malignancies which is considered to be an oncogene [30].

In this study, Jurkat cells were treated with DOX to induce apoptosis, decreased cell viability, and cell cycle arrest. Compared with negative control, knockdown of DEK promoted DOX-induced cell apoptosis while further reducing S-phase cells and cell proliferation of Jurkat cells with DOX, accompanied by significant alterations in the expression of apoptosis- and cell cycle-related genes. DEK silencing has no effect on TP53-related apoptosis and CDKN2A-induced senescence in Jurkat cells with DOX treatment. Therefore, DEK overexpression may inhibit the activity of TP53 and CDKN2A in Jurkat cells through alternative mechanisms. DEK acts as a transcriptional corepressor to inhibit NF-*κ*B signaling, and NF-*κ*B can participate in the apoptosis process of malignant hematopoietic cell lines by acting on CDKN1A [28]. CDKN1A effectively inhibits cyclins with direct roles in G1/S transition, including CDK2, CDK3, CDK4, and CDK6, but it inhibits other known CDKs poorly [11, 12]. Therefore, further research is needed to determine whether DEK acts on CDKN1A in Jurkat cells through NF-*κ*B.

Apoptosis is a complex biological process, and chemotherapy drugs are often used to kill tumor cells to treat tumors. With the widespread application of anticancer drugs, dysregulation of apoptotic pathways has been shown to play an irreplaceable role in chemoresistance. Antiapoptotic protein BCL2L1 regulates apoptotic cell death through Bcl-2. Increased expression of BCL2L1 is associated with chemoresistance in T-ALL [31]. Consistent with our results, knockdown of DEK attenuated the BCL2L1 expression of Jurkat cells, and the effect was more pronounced with DOX. These results suggest that DEK silencing enhances the sensitivity of Jurkat cells to chemotherapeutic drugs.

Caspase-3 is a well-known proapoptotic marker. Proapoptotic caspase-3 is frequently activated during apoptosis. DEK silencing induces apoptosis of tumor cells by activation of caspase-9 and subsequent cleavage and activation of procaspase-3, which then cleaves different cellular endogenous substrates leading to cell death [32, 33]. Therefore, DEK silencing may enhance DOX-induced apoptosis by activating the mitochondrial pathway through activating caspase-9 and then caspase-3 in Jurkat cells. Consistent with the *in vitro* data, knockdown of DEK also suppressed the growth of Jurkat cell-derived tumors in mouse model, suggesting that DEK is a promising therapeutic target in T-ALL treatment.

In brief, the deletion of DEK under DOX treatment leads to the overexpression of caspase-3 and the downregulation of BCL2L1, indicating its role in regulating cell apoptosis; the level of CDK6 decreases, and the expression of CDKN1A increases, indicating its role in regulating cell cycle. These results indicate that the inhibition of DEK expression combined with DOX treatment is a possible therapeutic strategy for T-ALL. In general, all these data suggest that DEK silencing in T-ALL cells increases their sensitivity to DOX and may work as a novel therapeutic target to T-ALL.

## 5. Conclusion

In summary, DEK is highly expressed in Jurkat cells and promotes cell proliferation and colony formation in vitro. DEK silencing may promote DOX-induced cell apoptosis and cell cycle arrest, thus increasing the sensitivity of Jurkat cells to DOX treatment. Although the underlying mechanisms and effects of DEK on normal cells require further study, our results suggest that knockdown of DEK is a novel, potential therapeutic approach to overcome DOX resistance in T-ALL treatment.

## Data Availability

The data that support the findings of this study are available from the corresponding author upon reasonable request.

## Supplementary Materials

Supplementary Materials Supplemental Figure S1: DEK is highly expressed in tumor cell lines.
