# Supplementary material for: Inhibition of DEK Enhances Doxorubicin-Induced Apoptosis and Cell Cycle Arrest in T-Cell Acute Lymphoblastic Leukemia Cells
Source: Dis Markers. 2022 Jun 20;2022:9312971. doi: 10.1155/2022/9312971 (PMC9236779; doi:10.1155/2022/9312971)
Supplement: Supplementary Materials — Supplemental Figure S1: DEK is highly expressed in tumor cell lines. [file 9312971.f1.docx]

**Supplemental Figure S1**


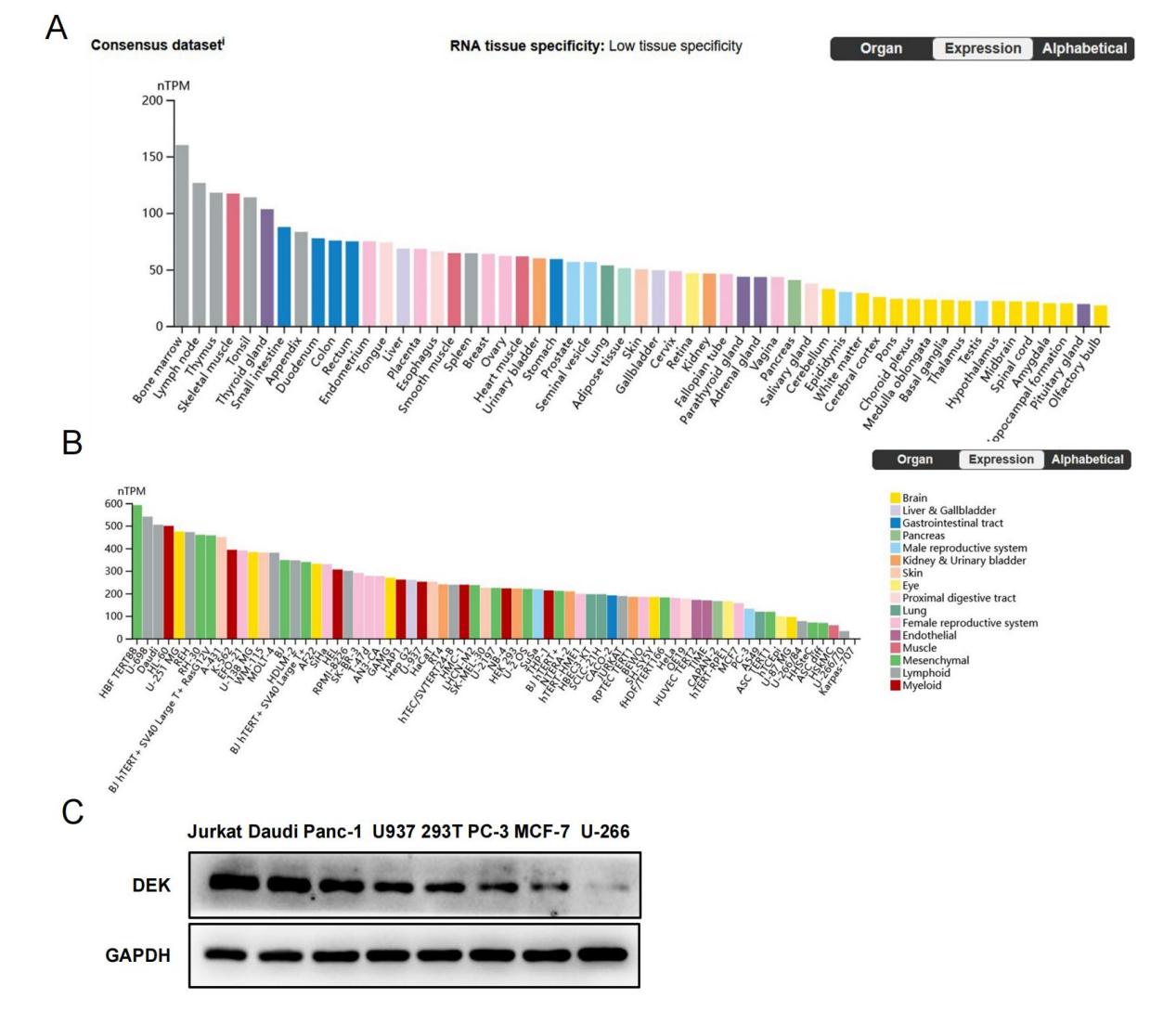


**Supplemental Figure S1：DEK is highly expressed in tumor cell lines.** (A) The comparison of the level of DEK mRNA transcripts in different tissues from the the human protein analysis. (B) Comparison of the expression level of DEK in different cell lines from the human protein analysis. (C) The DEK expression level of tumor cells.
